# Medical College of Tennessee

**Published:** 1841-08

**Authors:** 


### MEDICAL SOCIETY OF TENNESSEE.

In a former number, a fuller notice of the doings of this society, at its late meeting in May, was promised, and as a fulfilment of that promise, we have since devoted much of our space to the papers read on that occasion, deeming this the most satisfactory account that we could present. It is not, we confess, without some misgivings, that the first article in this number is published, and our readers must find our apology for it in the fact, that the very respectable society before which it was delivered requested its publication. It is our purpose to make the Journal as practical as we are able, and such a discourse, we are aware, however interesting the great topic of which it treats, cannot lay claims to this character. Even the request of the society we might have felt ourselves at liberty to disregard, if it had not been backed by a scarcity of matter which left us but little choice in the selection of articles. Our contributors, we are sure, will not often allow us to offer the latter apology. Y.

